# Predicting precursors of plant specialized metabolites using DeepMol automated machine learning

**DOI:** 10.1515/jib-2024-0050

**Published:** 2025-03-20

**Authors:** João Capela, João Cheixo, Dick de Ridder, Oscar Dias, Miguel Rocha

**Affiliations:** Centre of Biological Engineering, University of Minho, 4710-057, Braga, Portugal; Bioinformatics Group, Department of Plant Sciences, Wageningen University and Research, Wageningen, The Netherlands; LABBELS – Associate Laboratory, Braga/Guimarães, Portugal

**Keywords:** machine learning, plant specialized metabolites, biosynthesis

## Abstract

Plants produce specialized metabolites, which play critical roles in defending against biotic and abiotic stresses. Due to their chemical diversity and bioactivity, these compounds have significant economic implications, particularly in the pharmaceutical and agrotechnology sectors. Despite their importance, the biosynthetic pathways of these metabolites remain largely unresolved. Automating the prediction of their precursors, derived from primary metabolism, is essential for accelerating pathway discovery. Using DeepMol’s automated machine learning engine, we found that regularized linear classifiers offer optimal, accurate, and interpretable models for this task, outperforming state-of-the-art models while providing chemical insights into their predictions. The pipeline and models are available at the repository: https://github.com/jcapels/SMPrecursorPredictor.

## 1 Introduction

Gene duplication events have played a key role in shaping plant evolution, contributing to the expansion of gene functions and variability in gene content [1]. This genetic diversity is particularly noticeable in the evolution of lineage-specific specialized metabolites (SMs)[2], which serve to protect plants from both biotic and abiotic stressors, while also attracting and exploiting external agents such as pollinators [3]. The enzymes responsible for SM biosynthesis form distinct metabolic pathways, generating a wide range of compounds that hold significant value in industry, being widely used in agricultural, cosmetic and pharmaceutical products [4].

Despite their importance, the pathways and genes responsible for the biosynthesis of these SMs remain largely uncharted [3]. Even for the model plant *Arabidopsis thaliana*, nearly half of its genes still lack an assigned Gene Ontology term, possibly due to convergent evolution events, where unrelated enzymes from distant lineages with different sequences utilize common substrates to produce similar compounds [5], 6]. However, it is well understood that all SMs are synthesized through biosynthetic routes that utilize compounds derived from primary metabolic processes as building blocks [7]. These precursor compounds arise from key primary metabolic pathways, including photosynthesis, glycolysis, the citrate cycle, the shikimate pathway, mevalonate and the DOXP/MEP pathway [8], which provide the foundation for SM production.

Alkaloids are synthesized from amino acids, which provide the essential amine groups for their structure. These amino acids and various other components such as terpenoids, polyketides, and fatty acids contribute to alkaloids’ diverse structures [9].

For terpenoids, the isoprene rule states that their fundamental building blocks are the universal five-carbon precursors isopentenyl diphosphate (IPP) and dimethylallyl diphosphate (DMAPP) [10], 11]. The condensation of these two precursors leads to the formation of key prenyl diphosphate intermediates, such as Geranyl Diphosphate (GPP), Farnesyl Diphosphate (FPP), and Geranylgeranyl Diphosphate (GGPP), which serve as starting points for the synthesis of diverse terpenoid structures [10], 12].

Another key group of plant-specialized metabolites is the phenylpropanoid class. The shikimate pathway in plants acts as the entry point for their production by synthesizing the amino acid phenylalanine. The enzyme phenylalanine ammonia-lyase (PAL) then converts phenylalanine into cinnamic acid, initiating the phenylpropanoid biosynthetic pathway [8].

In 2019, Eguchi and colleagues introduced a Molecular Graph Convolutional Neural Network (MGCNN) specifically designed for the multi-label prediction of alkaloid precursors [13]. This model leverages atomic information and implements a “message passing” strategy, where data about neighbouring atoms is transmitted via the edges in the molecular graph. By aggregating and updating information at each atom, the network learns atom-level representations that capture the surrounding chemical environment. While the model outperformed traditional approaches like basic Neural Networks (NN) and Random Forests (RF) that were trained with Extended Connectivity Fingerprints (ECFP), the use of accuracy as the evaluation metric raises concerns given its limited suitability for unbalanced, multi-label datasets. Additionally, MGCNN produces predictions that lack interpretability, offering no insights into the key structural features driving the outcomes. Lastly, the study’s scope was restricted to alkaloids.

The present study seeks to address these limitations by exploring more suitable metrics for multi-label classification in unbalanced datasets and broadening the focus to include terpenoids, phenylpropanoids, glucosinolates, and benzoxazinoids. Furthermore, we utilized DeepMol’s automated machine learning (AutoML) module [14] to identify the optimal model for this specific task. This model was applied to predict the precursors of all plant compounds in LOTUS-DB [15], allowing for a critical analysis of the main prediction patterns across different compound classes on a larger scale. To demonstrate how our method offers improvements over the state-of-the-art, we compared it not only with MGCNN, but also with NeuralNPFP, a fingerprint specifically designed for Natural Products (NP), as well as other fingerprints that have been effective in classifying NPs by pathway, class, and subclass [16]. Additionally, we included MinHashed fingerprints (MHFP), which have shown promise for representing NPs in supervised bioactivity prediction tasks [17]. Finally, we aim to provide a method that offers interpretability regarding predictions.

## 2 Materials and methods

### 2.1 Data

This study employed a manually curated dataset consisting of 566 alkaloids and their biosynthetic precursors, sourced from scientific literature and the Kyoto Encyclopedia of Genes and Genomes (KEGG), as described by Eguchi et al. [13]. The dataset features 17 precursors, including 10 amino acids, one aromatic acid (anthranilate), five terpenoids, and indole-3-glycerol phosphate (IGP).

To further expand this dataset, we extracted the biosynthetic pathways for terpenoids, phenylpropanoids, glucosinolates, and benzoxazinoids from KEGG. These pathways were converted into directed acyclic graphs, with compounds represented as nodes and reactions as edges, and we employed depth-first search to identify precursors.

For terpenoids, the identified precursors included GPP (precursor of monoterpenoids [18]), GGPP (precursors of diterpenoids [18] and carotenoids [19]), FPP (precursors of sesquiterpenoids and triterpenoids [18]), and campesterol (precursor of brassinosteroids [20]).

The precursors for phenylpropanoids included L-phenylalanine, the universal starter for all phenylpropanoids, p-Coumaroyl-CoA (precursors of flavonoids and stilbenoids [8]), naringenin and liquiritigenin (isoflavonoids), as well as apigenin and kaempferol (flavones and flavonols [21]), and pelargonin, cyanidin, and delphinidin (anthocyanins [21]).

**Figure 1:**
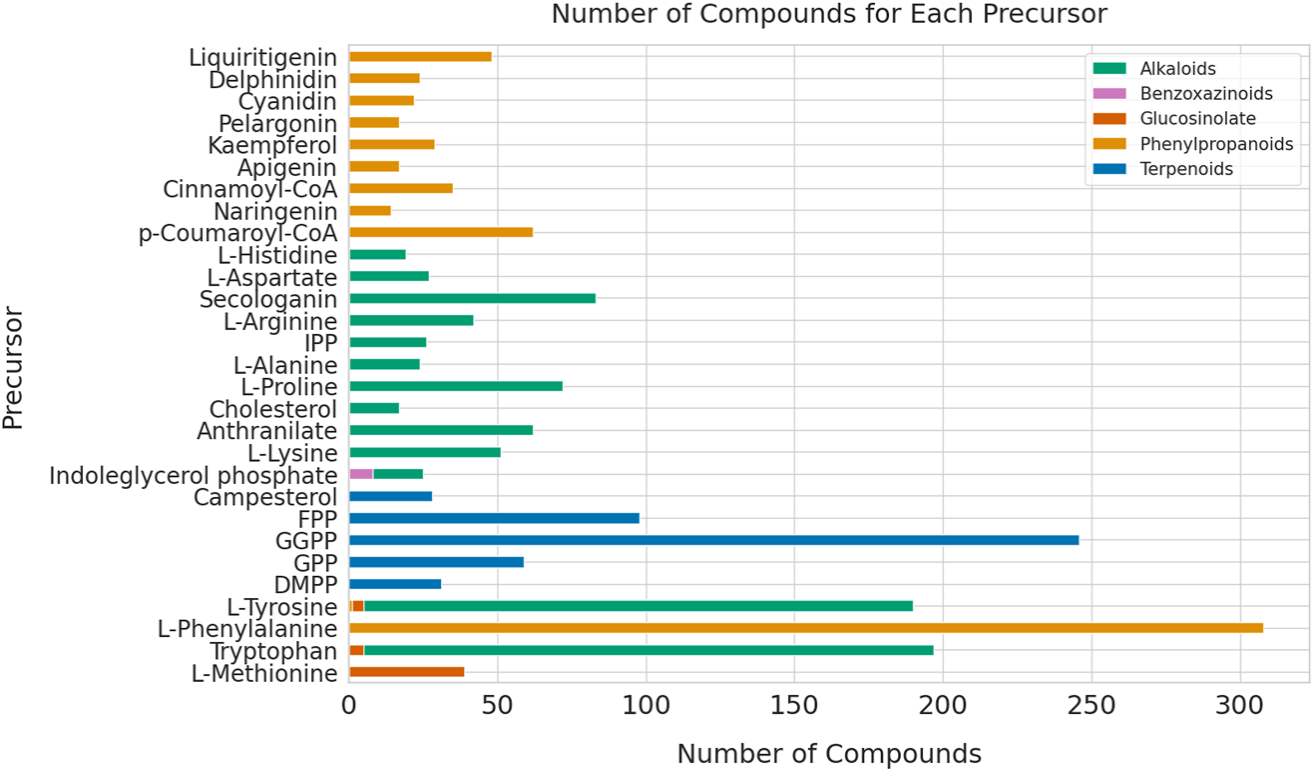
Number of compounds associated with each precursor. Note that one compound can have more than one precursor.

The precursors for glucosinolates are amino acids [22], which are already included in the dataset, while the precursor for benzoxazinoids is IGP [23]. Figure 1 illustrates the number of compounds associated with each precursor.

**Figure 2:**
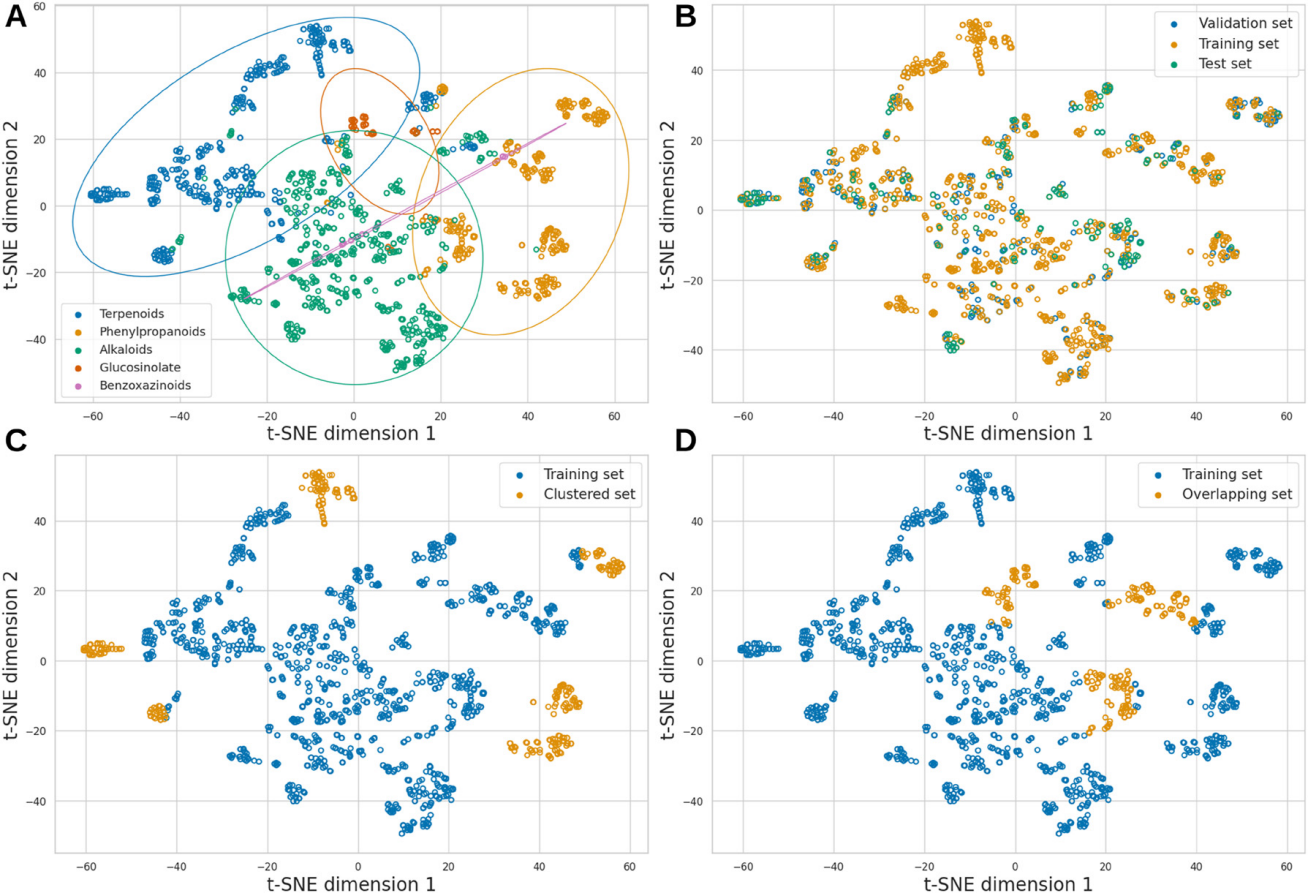
Chemical space of the dataset visualised using t-SNE. (A) Natural product classes. (B) Training, validation and test sets. (C) A training and test set with compounds from distant similarity clusters. (D) A training and test set where compounds from different natural product classes overlap in similarity.

The dataset was divided into training (70 %), validation (20 %), and test (10 %) sets. Figure 2 shows clusters of compounds grouped by their chemical similarities using t-distributed Stochastic Neighbor Embedding (t-SNE) from a similarity matrix (Supplementary Material), positioning compounds with greater chemical similarity closer together, while those with significant differences are placed further apart. Figure 2A and B show the distribution of compounds by chemical class and dataset, respectively.

Additionally, the dataset was split into two alternative ways to evaluate model robustness under more challenging training-test conditions. First, we selected more distant compound clusters (Figure 2C), ensuring at least minimal precursor representation by including a few compounds in the training set; otherwise, the model could not predict that specific precursor. Second, compounds that are highly similar but belong to different NP classes were excluded from the training set and included in the test set, as illustrated in Figure 2D. This configuration creates a challenging prediction task because, despite the molecules’ high similarity, they originate from different precursors.

### 2.2 Metrics

The metrics used include the F1 score, macro F1 score (mF1), recall, macro recall (mRecall), precision, and macro precision (mPrecision), each offering insights into different aspects of model performance.

The mF1 score is calculated as the harmonic mean of precision and recall for each label individually, and these values are then averaged to produce a single score for the entire dataset. A similar approach is used for mRecall and mPrecision, where the averages of precision and recall across labels are computed. The exact formulations can be found below.

(1) $$\text{Precision}=\frac{\text{True Positives}}{\text{True Positives}+\text{False Positives}}$$

(2) $$\text{Recall}=\frac{\text{True Positives}}{\text{True Positives}+\text{False Negatives}}$$

(3) $$\text{F}1=\frac{2\cdot \text{Precision}\cdot \text{Recall}}{\text{Precision}+\text{Recall}}$$

(4) $$\text{mPrecision}=\frac{1}{N}\overset{N}{\underset{i=1}{\sum }}\frac{{\text{True Positives}}_{i}}{{\text{True Positives}}_{i}+{\text{False Positives}}_{i}}$$

(5) $$\text{mRecall}=\frac{1}{N}\overset{N}{\underset{i=1}{\sum }}\frac{{\text{True Positives}}_{i}}{{\text{True Positives}}_{i}+{\text{False Negatives}}_{i}}$$

(6) $$\text{mF}1=\frac{1}{N}\overset{N}{\underset{i=1}{\sum }}\frac{2\cdot {\text{Precision}}_{i}\cdot {\text{Recall}}_{i}}{{\text{Precision}}_{i}+{\text{Recall}}_{i}}$$

Where *N* represents the total number of labels, with Precision_*i*_ and Recall_*i*_ indicating the precision and recall for label *i*, respectively. True Positives_*i*_ are the true positive predictions for label *i*, and False Negatives_*i*_ are the missed predictions for label *i*. Finally, the False Positives_*i*_ represent the incorrect positive predictions.

### 2.3 Automated machine learning

Our experimental framework utilized the DeepMol AutoML engine (version 1.1.5) [14] to train all classifiers for multi-label classification available in *scikit-learn* version 1.2.0, employing molecular fingerprints (FPs) and descriptors as inputs on the training set, and evaluating their performance on the validation set. The models implemented include ridge classifiers, decision trees, random forests, and k-nearest neighbours, among others.

AutoML streamlines ML tasks by automating the search for the optimal pipeline, which integrates a feature set, feature selection, and classifier. The main goal during the optimization process was to maximize the mF1 score on the validation set. We employed the Tree-structured Parzen Estimator (TPE) algorithm over 500 trials to determine the most appropriate hyperparameters and methods. Upon completing these trials, we identified the best pipeline and subsequently retrained it using the combined training and validation data to assess its performance on the test set.

### 2.4 Fingerprints for comparison

To benchmark our method, we compared it against several established molecular fingerprints (FPs) that have shown promise for NP classification and bioactivity prediction.

The following fingerprints were considered:

– MinHashed Fingerprints (MHFP) is a string-based FP algorithm that operates similarly to circular fingerprints, but instead of using atom identifiers, it uses SMILES substrings as fragment identifiers. These identifiers are then hashed and stored in a fixed-size vector using the MinHash algorithm, as shown by Probst et al. [24]. A recent study also highlighted MHFP’s effectiveness in NP bioactivity prediction [17].
– Neural Natural Product Fingerprints (NeuralNPFP) is a neural network-based molecular representation specifically designed for NPs. This FP encodes implicit NP-relevant information while maintaining details about their chemical space by training neural networks to differentiate between natural and synthetic compounds [25].
– NPClassifier Fingerprints are a variation of Morgan FPs designed for NP classification, where non-negative integers represent the count of repeated atomic substructures (or molecular fragments) rather than binary values. Morgan fingerprints represent molecular structures by encoding the substructures within a circular region around each atom into a fixed-length bit vector. These substructures are hashed into unique identifiers, with corresponding bits in the vector set to indicate the presence of specific atomic environments. The fingerprint’s size and detail are controlled by adjusting the radius, which defines the neighbourhood size around each atom. NPClassifier FPs combine three Morgan FPs, each with different radii (0, 1, 2), to capture structural information at varying distances [16].

This set of FPs highlights the diversity of approaches in molecular representation, from neural network-based embeddings to substructure counting, which provides a strong foundation for assessing the strengths of our method.

To ensure a fair comparison, we implemented each FP in DeepMol and used its optimization framework to identify the best combination of methods and models, while keeping the same fingerprint consistent across all trials. For each FP, we conducted 50 optimization trials using the TPE algorithm, following the standard AutoML approach in DeepMol.

### 2.5 Statistical methods

We employed the Wilcoxon Signed-Rank (WSR) Test to evaluate differences in the metric values between the models for all the test sets. In this context, the samples consist of the metric values obtained by two models for each label within the multi-label classification framework. The objective is to determine the significance of any difference in the median value of a metric across all labels. This hypothesis test is formulated as follows. Given metric values for two models across *n* labels, *m*_1*i*_ and *m*_2*i*_, calculate the differences *d*_*i*_ = *m*_1*i*_ − *m*_2*i*_ for each label *i*. For these differences, ignore *d*_*i*_ = 0 and rank the absolute differences |*d*_*i*_|, assign ranks *R*_*i*_ and compute $W^{+}=\sum_{d_{i}>0}R_{i}$ and $W^{-}=\sum_{d_{i}<0}R_{i}$ , the test statistic *W* is defined as *W* = min(*W* ^+^, *W* ^−^). A p-value is calculated as the probability of observing a value of *W*_*ref*_, determined by a reference distribution under the null hypothesis, as extreme as or more extreme than the observed value (*W*). The null hypothesis is that there are no significant differences between the median metric values between the two models. We considered a p-value lower than 0.05 sufficient to reject the null hypothesis.

Additionally, we applied the same test to each pair of models for the experimental setup outlined in [13], where cross-validation is conducted solely for predicting the precursors of alkaloids. This hypothesis test is formulated as follows. Given two models evaluated across *n* labels and *r* folds, resulting in performance metrics *m*_*Aij*_ and *m*_*Bij*_ for models *A* and *B* respectively, for each label *i* and fold *j*, we perform the following steps: calculate the differences *d*_*ij*_ = *m*_*Aij*_ − *m*_*Bij*_, rank the absolute differences |*d*_*ij*_|, and apply the Wilcoxon Signed-Rank Test as explained above.

### 2.6 Precursor predictions for LOTUS-DB plant compounds

After selecting the best model, we retrained it with the collected data (train, validation and test sets). Next, we downloaded all the data from LOTUS-DB and filtered the compounds annotated as plant compounds. We then predicted the precursors for all these compounds using the retrained model and analyzed the main patterns of predictions by class of compounds, afterwards.

## 3 Results and discussion

### 3.1 AutoML consistently outperform MGCNN and NP-focused fingerprints

**Figure 3:**
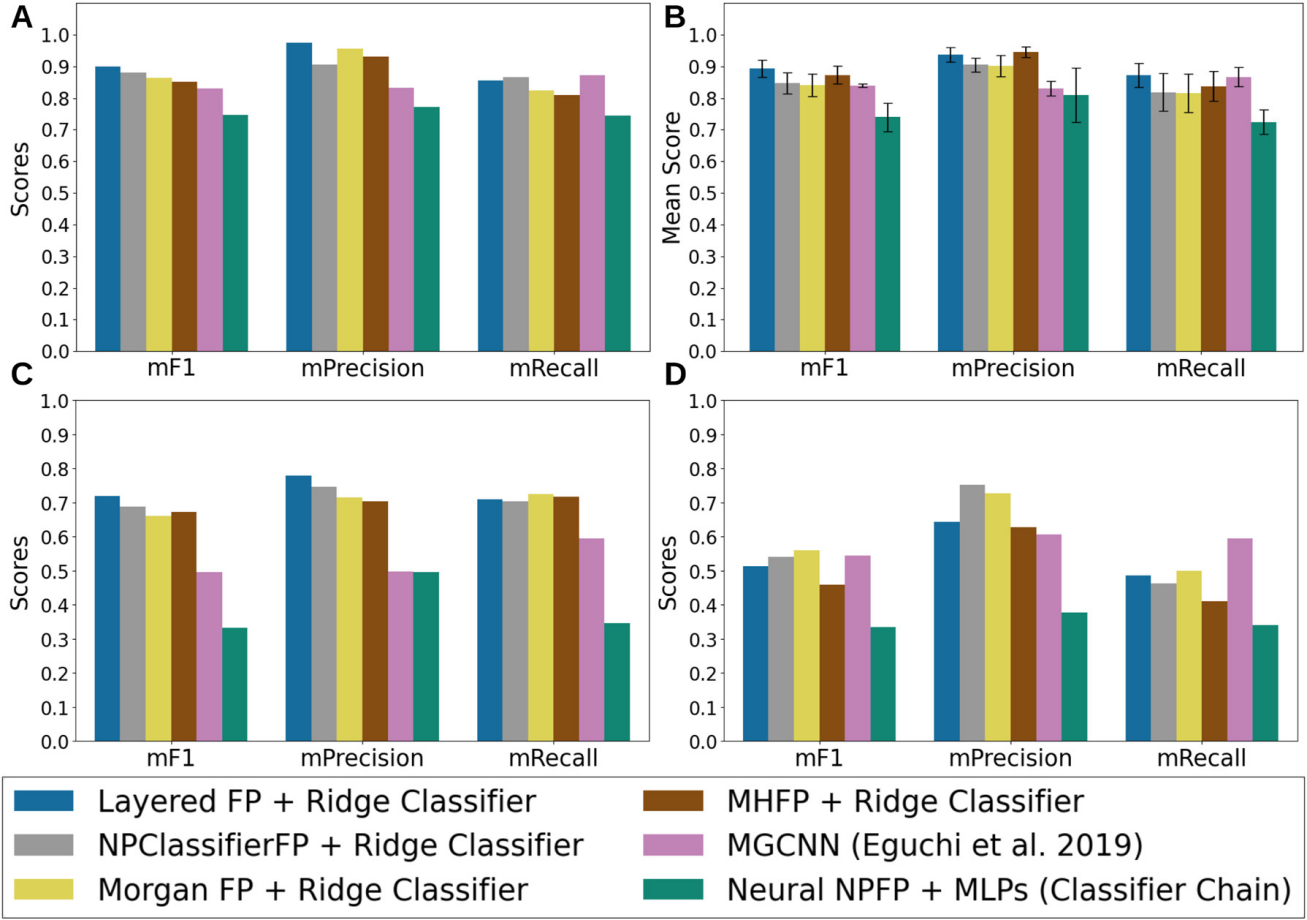
Results of each model evaluated on four different settings (see methods). (A) The test set originated from the stratified split. (B) Cross-validation datasets for predicting the precursors of alkaloids from the Eguchi et al. 2019 paper. (C) The distant similarity clustered compounds dataset. (D) the overlapping natural product classes dataset.

Figure 3 provides a comparative analysis of several classification models across three metrics: macro F1-score (mF1), macro Precision (mPrecision), and macro Recall (mRecall).

The models were selected based on the outcomes of an AutoML search, where Ridge classifiers using Morgan FPs and Layered FPs emerged as the top performers in terms of model and feature extraction methods, respectively. Among the models, the standout performer was a Ridge classifier trained on Layered FPs. The Layered FPs are molecular FPs that capture multiple structural attributes of a molecule using a multi-layered approach, where each layer captures different molecular features, including topology, bond order, atom types, ring presence and sizes, and aromaticity. The construction process involves generating a multi-layered hash for each molecular subgraph to set bits in the fingerprint, allowing for flexible representation. On top of this, the AutoML engine from DeepMol improved this model’s validation results by applying feature selection, specifically filtering out features with a variance below 0.0628, which was identified as the optimal threshold by the AutoML. The approach that combines Layered FPs, a variance threshold filter, and a Ridge classifier is called the *Layered FP*+*Ridge Classifier*. In addition, we evaluated a Ridge classifier trained with Morgan FPs, known for their straightforwardness in mapping molecular substructures directly to specific FP bits. Similar performance patterns were observed for NPClassifier FP and MHFP, where Ridge classifiers achieved the best results. However, for the Neural NPFP, a classifier chain model leveraging a Multi-Layer Perceptron (MLP) as the base estimator delivered the top performance.

*Layered FP*+*Ridge Classifier* delivered the best performance on the test dataset (Figure 3A), achieving an mF1 score of 0.898 and an mPrecision of 0.975. This classifier demonstrated a well-balanced trade-off between mPrecision and mRecall, with a slight bias towards mPrecision. Notably, this model exhibited significant improvements over the MGCNN model [13]. It achieved markedly higher F1 and precision scores than MGCNN, with statistical significance (*p* − *value*_*F*1_ < 0.05, *p* − *value*_*precision*_ < 0.01, in a WSR test, see Supplementary Material and Methods). While other models, such as *NPClassifierFP*+*Ridge Classifier*, *MHFP*+*Ridge Classifier*, and *Morgan FP*+*Ridge Classifier*, also outperformed MGCNN in terms of mF1 score, the *Layered FP*+*Ridge Classifier* was the only one to achieve a statistically significant improvement. As depicted in Figure 3A, MGCNN yielded a marginally higher mRecall (0.871) than the other models, though this difference was statistically significant only in comparison to *MHFP*+*Ridge Classifier* (*p* − *value*_*recall*_ < 0.05, in a WSR test) and *NeuralNPFP*+*MLPs* (*p* − *value*_*recall*_ < 0.01, in a WSR test). The pipeline utilizing NeuralNPFP underperformed across all metrics when compared to the other approaches.

The *Layered FP*+*Ridge Classifier* with the Ridge classifier outperformed MGCNN in predicting nearly all precursors, except cholesterol, campesterol, and FPP. This indicates that MGCNN exhibits slightly stronger predictive capabilities for steroid, sesquiterpenoid, and triterpenoid precursors (for further details, refer to the Supplementary Material). When focusing specifically on the alkaloids dataset from Eguchi et al. [13] (Figure 3B), *Layered FP*+*Ridge Classifier* consistently outperformed the state-of-the-art MGCNN across all metrics, with statistically significant improvements in mF1 and mPrecision. Although the *MHFP*+*Ridge Classifier* achieved a higher mPrecision than the *Layered FP*+*Ridge Classifier*, this difference was not statistically significant, and the MHFP model underperformed in terms of mRecall and mF1. Furthermore, the *Layered FP*+*Ridge Classifier* was the only approach that surpassed MGCNN across all metrics.

These performance trends persisted with the clustered dataset (Figure 3C), where the Ridge classifiers consistently outperformed MGCNN across all metrics. However, the differences were not statistically significant, likely due to the small number of labels evaluated (13) and the high variability in label scores across different methods (see Supplementary Material). The only statistically significant differences were observed when comparing all Ridge classifiers to the *NeuralNPFP*+*MLPs* pipeline.

In the overlapping dataset (Figure 3D), the *Morgan FP*+*Ridge Classifier* and MGCNN models showed the best performance, although their advantage was slight and not statistically significant, except in precision scores, where the *Morgan FP*+*Ridge Classifier* significantly outperformed the *Layered FP*+*Ridge Classifier*. All models showed a substantial improvement over the *NeuralNPFP*+*MLPs* in terms of F1 scores. While other models exhibited lower precision scores, these differences were not statistically significant due to the large variability across labels (more details can be found in the Supplementary Material).

The ML pipelines discovered by DeepMol AutoML demonstrated clear advantages over models utilizing Natural Product-focused fingerprints and the state-of-the-art tool MGCNN. While the Natural Product-focused representations such as NPClassifierFP and MHFP had isolated strengths in certain metrics, they generally underperformed compared to the Ridge classifiers using Layered and Morgan FPs. These findings highlight the importance of feature selection and optimization, as facilitated by DeepMol’s AutoML framework, in achieving state-of-the-art performance in molecular classification tasks.

### 3.2 Predictions for LOTUS-DB compounds show consistent patterns

Next, we predicted the precursors for all plant compounds in LOTUS-DB to annotate them and provide comprehensive insights. We also conducted a large-scale analysis of the patterns in our predictions across all the 190,273 compounds.

**Figure 4:**
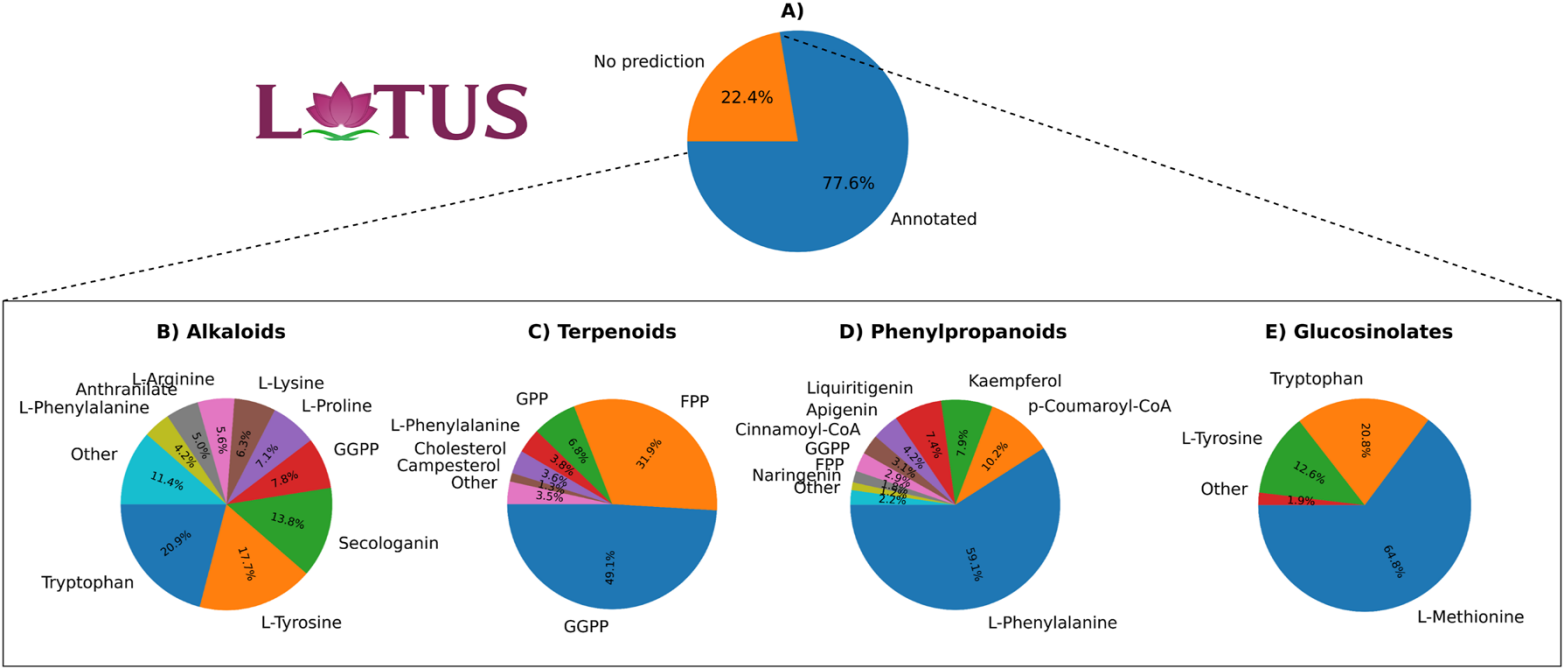
Predictions for LOTUS-DB using *Layered FP*+*Ridge Classifier*. The top pie chart illustrates the percentage of annotated and unannotated plant compounds in LOTUS-DB. A majority (77.6 %) of the compounds are annotated, while 22.4 % remain unannotated. The bottom four pie charts show the percentage of predictions for each precursor. Each chart represents a different subset of compound classes.

The pie charts in Figure 4 offer insights into the predictions made by the *Layered FP*+*Ridge Classifier* for plant compounds in LOTUS-DB [15], a comprehensive database of NPs with taxonomic details and chemical class ontologies. This database enables the filtering of compounds based on their chemical class and the organisms responsible for their production, facilitating our further analysis.

The top chart (Figure 4A) illustrates that our tool predicts the precursors of 77.6 % of plant compounds in LOTUS-DB, leaving 22.4 % unannotated. These unannotated compounds likely stem from primary metabolism or have precursors not included in the current dataset. While compounds from primary metabolism probably represent only a small portion of this group, the majority might consist of metabolites with precursors outside our current scope. For example, polyketides are synthesized from precursors like acetyl-CoA, malonyl-CoA, and propionyl-CoA, none of which were included in our dataset, as plant polyketide production was not a focus of this study. Expanding the dataset to include additional precursors, especially those involved in the biosynthesis of polyketides and other important plant metabolite classes, will be an important next step.

The bottom charts display the percentage of predicted precursors involved in the biosynthesis of alkaloids, terpenoids, phenylpropanoids, and glucosinolates. Benzoxazinoids were excluded from the analysis as this class of compounds was not in the database.

In the first chart (Figure 4B), which focuses on predictions for alkaloids, tryptophan is identified as the most prevalent precursor, accounting for 20.9 % of the predictions, followed by L-tyrosine at 17.7 %, and secologanin at 13.8 %. Other precursors contribute smaller percentages. The prevalence of amino acids, some terpenoids such as secologanin, and the aromatic acid anthranilate align well with expectations, as these are known precursors of alkaloids. However, the dominance of tryptophan, L-tyrosine, and secologanin could indicate a bias in the predictions, potentially caused by label imbalance, where overrepresented labels skew predictions in their favour (see Methods, Figure 1). Alternatively, it may reflect the actual distribution of compounds in the database, where a significantly larger proportion of compounds are produced from these most prevalent metabolites compared to others. Interestingly, despite the label imbalance, anthranilate did not appear as the 5th most common label as expected, even though it holds that position (as 5th most prevalent label for alkaloids) in our dataset (see Methods, Figure 1). Although GGPP was not included in the dataset as an alkaloid precursor, alkaloids are known to be hybrid structures that can incorporate terpenoids into their carbon skeletons. This observation suggests that the model has learned chemical patterns from terpenoids, enabling it to generalize and apply this knowledge to predict for other compound classes, even when specific precursors, like GGPP, are not explicitly present in the alkaloid dataset.

In the second chart (Figure 4C), which focuses on the precursors of terpenoids, GGPP is the most prevalent label, accounting for 49.1 % of the predictions, while FPP contributes 31.9 %. The contributions from other precursors are significantly smaller, with the third precursor (GPP) contributing 6.8 %. Another precursor, campesterol, had a lower prevalence compared to L-phenylalanine and cholesterol. The predictions involving L-phenylalanine are questionable, as terpenoids are not synthesized from this amino acid. Cholesterol, on the other hand, is a terpenoid, and despite not being explicitly included in the dataset as a terpenoid precursor, it is understandable that the model would make this prediction, especially when steroids are being analyzed. Once again, the overrepresented labels also appeared most frequently in the predictions.

The third chart (Figure 4D) focuses on the prediction of phenylpropanoid precursors. Unsurprisingly, the dominant precursor, responsible for 59.1 % of the predictions, was L-phenylalanine, followed by p-Coumaroyl-CoA at 10.2 % and Kaempferol at 7.9 %. Most of the other precursors were expected as they were assigned as phenylpropanoid precursors. FPP and GGPP were also predicted as precursors, which is questionable, as they are not known to be involved in phenylpropanoid biosynthesis. However, the fact that L-phenylalanine was predicted as a precursor for some terpenoids, and FPP and GGPP as phenylpropanoid precursors, suggests that these metabolites could be part of complex natural products, such as hybrid molecules that incorporate elements from both pathways. Once again, the most frequent predictions were associated with the overrepresented labels.

Finally, in the fourth chart (Figure 4E), focused on the production of glucosinolates, L-methionine accounts for 64.8 % of the predictions, with tryptophan contributing 20.8 % and L-tyrosine 12.6 %. These results are in accordance with the precursors defined for glucosinolates.

The predictions made for all plant compounds in LOTUS-DB highlight the effectiveness of our model in annotating a dataset of 190,273 compounds and revealing insights into the underlying biosynthetic pathways. The detailed breakdown of precursor predictions across various compound classes reveals clear patterns and interesting challenges. The model correctly identified common precursors such as tryptophan, L-phenylalanine, and GGPP, but also seems to have faced issues with overrepresented labels, which may have skewed certain predictions. For instance, in alkaloid biosynthesis, the high prevalence of tryptophan and L-tyrosine may reflect a bias in the data, while in terpenoid and phenylpropanoid classes, a few questionable predictions might indicate the complexity of natural product biosynthesis and the possibility of hybrid structures.

Overall, these results demonstrate the robustness of our predictive model, while also highlighting areas for future improvement, such as refining the handling of label imbalances and expanding the dataset to cover other biosynthetic pathways.

### 3.3 Ridge classifier can make and explain relevant predictions

Next, we aimed to evaluate the predictive capabilities of the *Morgan FP*+*Ridge Classifier* pipeline on several complex cases and analyze the significance assigned to specific features. This pipeline was selected for its ability to trace FP bits back to specific molecular structures, a feature that is not available with Layered FPs in the *Layered FP*+*Ridge Classifier*. We selected four compounds for this analysis: two monoterpenoid indole alkaloids recognized for their cryptic biosynthesis and two others with partially elucidated biosynthetic pathways.

**Figure 5:**
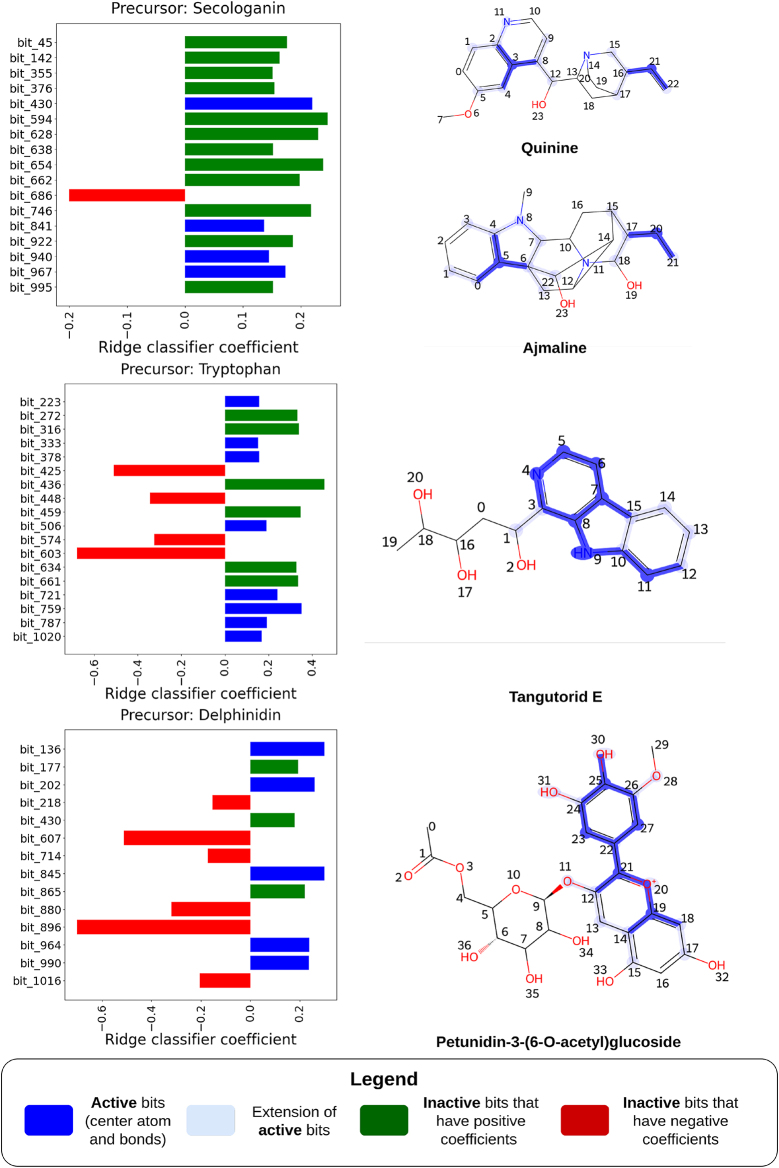
Predictions made by the *Morgan FP*+*Ridge Classifier* model and the importance given by the model to structural features in the compounds. On the left, bar plots show the top Ridge classifier coefficients, which correspond to bits in the FP. Red bars represent inactive negative coefficients, green bars show inactive positive bits and dark blue bars indicate active positive coefficients. The molecules subjected to the predictions are presented on the right, and the most important bits of the FP (represented in dark blue in each respective bar plot) are highlighted, where the extension of the bits (atoms and bonds within the radius defined by MorganFPs) is shaded by faded blue.

Figure 5 illustrates the relationship between the model coefficients of the *Morgan FP*+*Ridge Classifier* and the substructures of the compounds. These coefficients represent the weights assigned to each feature, defining the hyperplane that separates the labels. Each coefficient corresponds to a specific feature (or molecular substructure) and indicates the extent and direction (positive or negative) of its influence on the label prediction.

The carbon skeletons of the selected monoterpenoid indole alkaloids, quinine and ajmaline, are derived from both tryptophan and secologanin. Quinine, an alkaloid obtained from the Cinchona trees of the Rubiaceae family, has a long history as an antimalarial treatment [26]. Ajmaline, on the other hand, is an antiarrhythmic drug used in diagnosing Brugada syndrome, a rare type of arrhythmia [27].

The biosynthesis of these compounds begins with the condensation of tryptamine (derived from tryptophan) and secologanin, catalyzed by strictosidine synthase. Despite this, the substructures originating from secologanin largely disappear in the final structures, especially in the case of ajmaline. This suggests that predicting these molecules as derivatives of secologanin is challenging, given that the distinct features of secologanin may not be evident in the final chemical structure.

An experiment was conducted in which we identified the key substructures of secologanin and selected 18 molecules where these substructures largely disappear in the final structures. These molecules are still related to secologanin but with few of its substructures retained during biosynthesis, making them hard to annotate and link to the precursor. They were excluded from the training set. Despite this, secologanin was still predicted as a major precursor for these molecules, achieving a recall of 0.89, precision of 1.00, and an F1 score of 0.94, showing the model’s strong predictive ability.

Figure 5 presents the interpretability of the predictions for quinine. The most important features for predicting quinine primarily include the quinoline heterocyclic ring and a propene fragment. The high coefficient for the FP bit corresponding to the quinoline ring system may suggest an unknown step in the biosynthesis of quinine. It is believed that the quinoline structure found in cinchoninone arises from the rearrangement of cinchonaminal, which is thought to be involved in quinine’s biosynthesis [26]. Interestingly, the strong signal for the quinoline ring cannot be attributed to any significant presence of compounds with similar structures in the training set, as it did not contain such compounds; instead, these were all reserved for the test set. In contrast, the high coefficient for the propene fragment’s FP bit aligns with chemical intuition, as this fragment is the only part of the secologanin carbon skeleton retained in quinine [26].

For ajmaline, no substructure directly associated with secologanin was identified. However, rather than a propene fragment, a propane fragment exhibited a strong signal and played a crucial role in model prediction. This observation could be linked to a reduction event during the biosynthesis of ajmaline, where a propene fragment derived from the secoiridoid moiety of secologanin undergoes reduction by the 1,2-dihydrovomilenine 19,20(S)-reductase enzyme [27]. This reduction results in the propane moiety, which is highlighted in dark blue in Figure 5, further supporting the model’s predictive power in capturing biosynthetic subtleties.

The third compound featured in Figure 5, tangutorid E, is a *β*-carboline alkaloid found naturally in tomatoes and recently identified as a biomarker for human consumption of tomato juices [28]. *β*-carboline alkaloids are produced through a Pictet-Spengler reaction between indolethylamines, such as tryptophan-derived compounds, and aldehydes or *α*-ketoacids [29].

The model predicts that tangutorid E originates from tryptophan, with its *β*-carboline moiety playing a crucial role in this prediction. The presence of the indole structure and the *α*-amino group in tryptophan are key features influencing this outcome. This highlights the model’s focus on critical structural elements related to the biosynthesis of *β*-carboline alkaloids, emphasizing its ability to capture essential chemical patterns associated with these complex molecules.

The fourth compound is a phenylpropanoid produced by *Vitis vinifera*, Petunidin-3-(6-O-acetyl)glucoside, an anthocyanin discovered in the grapevine berry, which contributes to its blue colour. It is produced through the methylation of delphinidin glycosides [30], 31].

The model identifies delphinidin as its precursor and focuses on the structural features of anthocyanin precursors – pelargonidin, cyanidin, and delphinidin – that differ primarily by the number of hydroxyl groups attached to the benzene ring (atoms 22–27 in Figure 5). Delphinidin, for instance, contains three hydroxyl groups, while pelargonidin and cyanidin have one and two, respectively. The model assigns particular importance to the bits corresponding to these hydroxyl fragments to differentiate between the anthocyanin precursors.

Additionally, the model highlights the central pyran ring in its cationic form, which is a distinguishing feature for anthocyanin precursors, setting them apart from other phenylpropanoid precursor groups such as flavones, flavanols, and isoflavonoids. This emphasizes the model’s sensitivity to the key structural elements that define the biosynthetic pathways of anthocyanin compounds.

## 4 Conclusions

Our work extends the dataset of Eguchi and collaborators [13] to include terpenoid, phenylpropanoid, glucosinolate and benzoxazinoid precursors rather than only alkaloids. By leveraging DeepMol’s AutoML engine to select the optimal combination of ML models, feature extraction, and selection methods, we improved the prediction of not only alkaloid precursors but also those of all other compound classes. We proved that Ridge classifiers outperform the state-of-the-art model, MGCNN, across multiple datasets, offering both accuracy and interpretability. Additionally, the combination of features and models identified by DeepMol’s AutoML exceeds the performance of models trained with NP-focused FPs such as NeuralNPFP [25] and NP Classifier FP [16], or one of the most promising FPs for representing NPs for bioactivity prediction tasks (MHFP) [17], 24]. The structural features that influence model predictions are also closely aligned with the biosynthetic pathways leading to the final compounds, further validating the effectiveness of the model.

Upon predicting precursors for 190,273 plant metabolites from LOTUS-DB, we successfully predicted precursors for over three-quarters of the compounds, which is a strong result. However, this also highlights that certain metabolite classes, such as polyketides, were not predicted due to missing precursors in our dataset. Additionally, some compounds from general or primary metabolism, as expected, were not annotated. The prediction patterns in this large database showcase the robustness and efficiency of our model, which accurately predicted the expected biosynthetic precursors for various compound classes, like alkaloids. However, overrepresented labels posed challenges, potentially skewing some predictions.

Improvements to our method include expanding the dataset to include more precursors and addressing the issue of label imbalance. Future work will harness the strengths of this model to enhance pathway enumeration algorithms and retrobiosynthesis tools like BioNavi-NP [32] and READRetro [33], enabling the rapid identification of potential starting points and critical building blocks while excluding irrelevant precursor candidates. This approach will streamline the exploration of biosynthetic pathways, potentially accelerating the discovery and understanding of natural product biosynthesis.

## Supplementary Material

Supplementary Material Details

## References

[1] Panchy N, Lehti-Shiu M, Shiu SH (2016). Evolution of gene duplication in plants. Plant Physiol.

[2] Chae L, Kim T, Nilo-Poyanco R, Rhee SY (2014). Genomic signatures of specialized metabolism in plants. Science.

[3] Moore BM, Wang P, Fan P, Leong B, Schenck CA, Lloyd JP (2019). Robust predictions of specialized metabolism genes through machine learning. Proc Natl Acad Sci USA.

[4] Zhou F, Pichersky E (2020). More is better: the diversity of terpene metabolism in plants. Curr Opin Plant Biol.

[5] Bolger ME, Arsova B, Usadel B (2017). Plant genome and transcriptome annotations: from misconceptions to simple solutions. Briefings Bioinf.

[6] Pichersky E, Lewinsohn E (2011). Convergent evolution in plant specialized metabolism. Annu Rev Plant Biol.

[7] Hussein RA, El-Anssary AA (2019). Plants secondary metabolites: the key drivers of the pharmacological actions of medicinal plants. Herbal Medicine.

[8] Vogt T (2010). Phenylpropanoid biosynthesis. Mol Plant.

[9] Aniszewski T (2015). Alkaloids: chemistry, biology, ecology, and applications.

[10] Tholl D (2015). Biosynthesis and biological functions of terpenoids in plants. Biotechnol Isoprenoids.

[11] Kubeczka KH (2020). History and sources of essential oil research. Handbook of Essential Oils.

[12] Henry LK, Thomas ST, Widhalm JR, Lynch JH, Davis TC, Kessler SA (2018). Contribution of isopentenyl phosphate to plant terpenoid metabolism. Nat Plants.

[13] Eguchi R, Ono N, Hirai Morita A, Katsuragi T, Nakamura, Huang M (2019). Classification of alkaloids according to the starting substances of their biosynthetic pathways using graph convolutional neural networks. BMC Bioinf.

[14] Correia J, Capela J, Rocha M (2024). Deepmol: an automated machine and deep learning framework for computational chemistry. J Cheminf.

[15] Wang K, Deng J, Damaris RN, Yang M, Xu L, Yang P (2015). LOTUS-DB: an integrative and interactive database for Nelumbo nucifera study. Database.

[16] Kim HW, Wang M, Leber CA, Nothias LF, Reher R, Kang KB (2021). NPClassifier: a deep neural network-based structural classification tool for natural products. J Nat Prod.

[17] Boldini D, Ballabio D, Consonni V, Todeschini R, Grisoni F, Sieber SA (2024). Effectiveness of molecular fingerprints for exploring the chemical space of natural products. J Cheminf.

[18] Davis EM, Croteau R (2000). Topics in Current Chemistry.

[19] Shumskaya M, Wurtzel ET (2013). The carotenoid biosynthetic pathway: thinking in all dimensions. Plant Sci.

[20] Zullo MAT, Bajguz A (2019). The brassinosteroids family–structural diversity of natural compounds and their precursors. Brassinosteroids: plant growth and development.

[21] Liu W, Feng Y, Yu S, Fan Z, Li X, Li J (2021). The flavonoid biosynthesis network in plants. Int J Mol Sci.

[22] Sønderby IE, Geu-Flores F, Halkier BA (2010). Biosynthesis of glucosinolates–gene discovery and beyond. Trends Plant Sci.

[23] Frey M, Schullehner K, Dick R, Fiesselmann A, Gierl A (2009). Benzoxazinoid biosynthesis, a model for evolution of secondary metabolic pathways in plants. Phytochemistry.

[24] Probst D, Reymond JL (2018). A probabilistic molecular fingerprint for big data settings. J Cheminf.

[25] Menke J, Massa J, Koch O (2021). Natural product scores and fingerprints extracted from artificial neural networks. Comput Struct Biotechnol J.

[26] Trenti F, Yamamoto K, Hong B, Paetz C, Nakamura Y, O’Connor SE (2021). Early and late steps of quinine biosynthesis. Org Lett.

[27] Guo J, Gao D, Lian J, Qu Y (2024). De novo biosynthesis of antiarrhythmic alkaloid ajmaline. Nat Commun.

[28] Hövelmann Y, Jagels A, Schmid R, Hübner F, Humpf HU (2020). Identification of potential human urinary biomarkers for tomato juice intake by mass spectrometry-based metabolomics. Eur J Nutr.

[29] Herraiz T (2008). *β*-Carboline alkaloids. Bioact Compds Foods.

[30] Minnaar P, Van Der Rijst M, Hunter J (2022). Grapevine row orientation, vintage and grape ripeness effect on anthocyanins, flavan-3-ols, flavonols and phenolic acids: I. Vitis vinifera L. cv. Syrah grapes. OENO One.

[31] Ng J, Smith SD (2015). How to make a red flower: the combinatorial effect of pigments. AoB Plants.

[32] Zheng S, Zeng T, Li C, Chen B, Coley CW, Yang Y (2022). Deep learning driven biosynthetic pathways navigation for natural products with BioNavi-NP. Nat Commun.

[33] Kim T, Lee S, Kwak Y, Choi MS, Park J, Hwang SJ (2024). READRetro: natural product biosynthesis predicting with retrieval-augmented dual-view retrosynthesis. New Phytol.

