# Supplementary material for: Predicting precursors of plant specialized metabolites using DeepMol automated machine learning
Source: J Integr Bioinform. 2025 Mar 20;22(2):20240050. doi: 10.1515/jib-2024-0050 (PMC12569576; doi:10.1515/jib-2024-0050)
Supplement: Supplementary file 1 — Supplementary Material Details [file j_jib-2024-0050_suppl_001.pdf]

# Supplementary Material - Predicting Precursors of Plant Specialized Metabolites Using DeepMol Automated Machine Learning

João Capela[1], João Cheixo[1], and Dick de Ridder[2], Miguel Rocha[1,3], Oscar Dias[1,3]

[1] Centre of Biological Engineering, University of Minho, 4710-057, Braga, Portugal
[2] Bioinformatics Group, Department of Plant Sciences, Wageningen University and Research, Wageningen, the Netherlands
[3] LABBELS – Associate Laboratory, Braga/Guimarães, Portugal

## 1 Similarity matrix and t-SNE generation

A similarity matrix between all the Morgan fingerprints of the compounds in the whole dataset was generated to assess their similarity. The similarity function was the Tanimoto similarity index. A t-distributed Stochastic Neighbor Embedding (t-SNE) was created from this matrix to reduce dimensionality and for visualization.

## 2 AutoML results

Fig. 1 and 2 show the automatic machine learning model results. Fig. 1 shows the features used during the optimization and the mF1 score on the validation set for each trial. Morgan and layered fingerprints (FP) stood out as the best features. Fig. 2 shows the models trained and the mF1 scores obtained by each model on the validation set. The ridge classifiers stood out unequivocally.

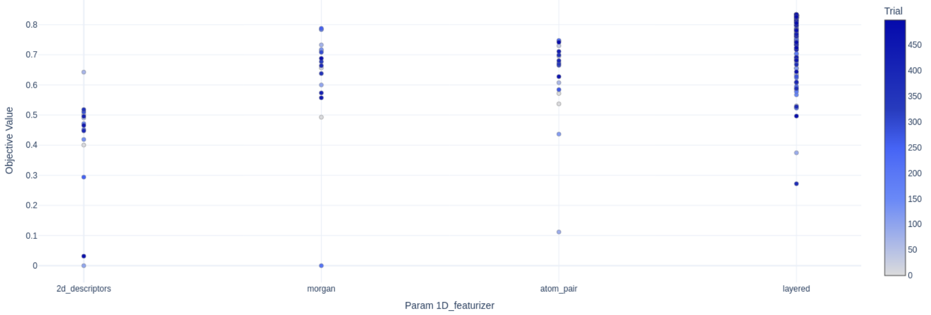


**Fig. 1.** mF1 score on the validation set in each trial per type of features.

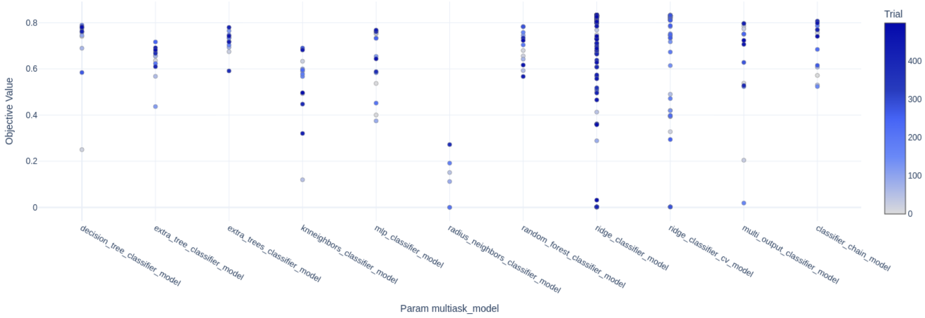


**Fig. 2.** mF1 score on the validation set in each trial per model.

# 3 Results per label

## 3.1 Label F1 scores - test dataset

Fig. 3 shows the F1 scores for each label and model.

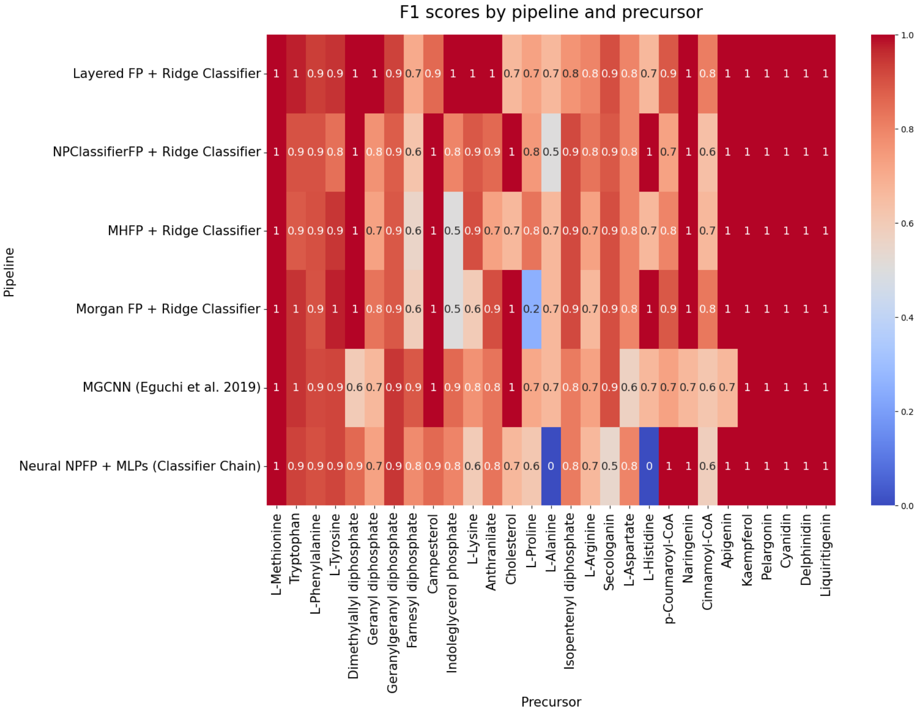


**Fig. 3.** F1 scores for each label and model

### 3.2 Label scores - clustered dataset

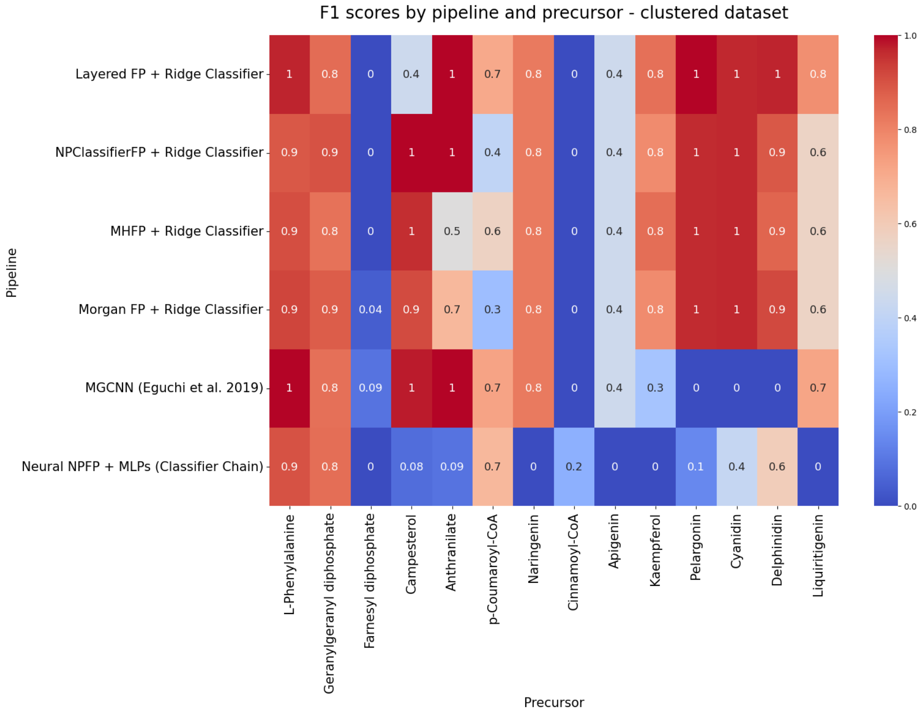


**Fig. 4.** F1 scores for each label and model

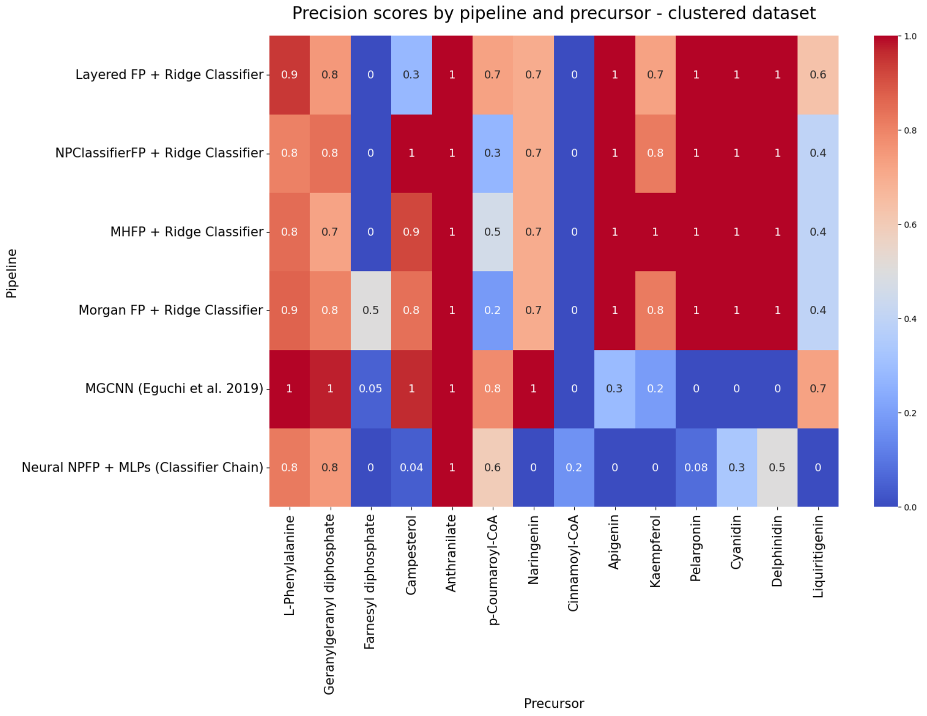


**Fig. 5.** Precision scores for each label and model

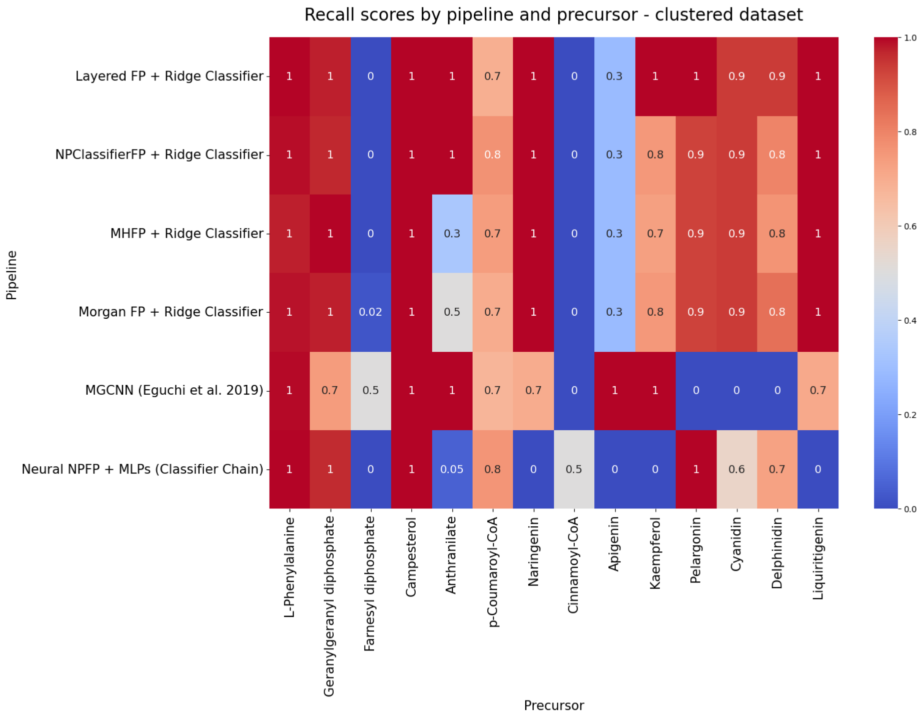


**Fig. 6.** Recall scores for each label and model

### 3.3 Label scores - overlapping dataset

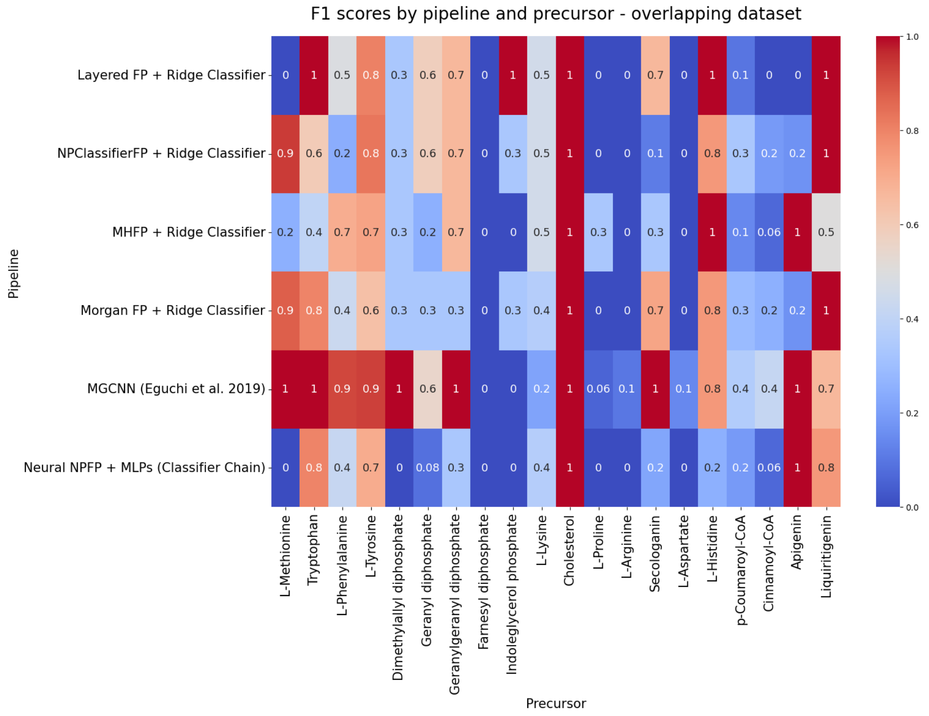


**Fig. 7.** F1 scores for each label and model

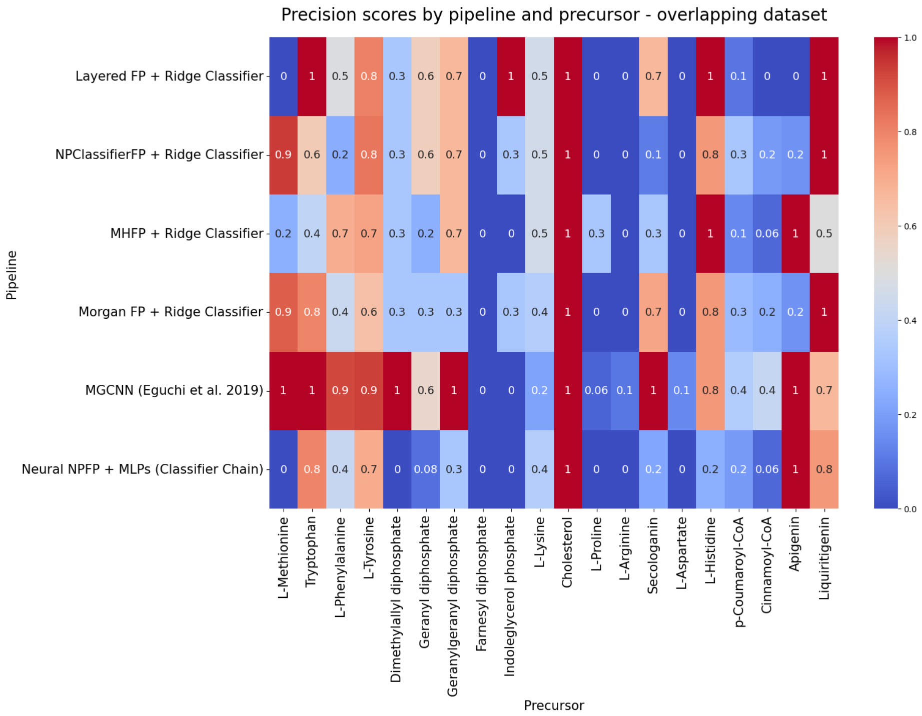


**Fig. 8.** Precision scores for each label and model

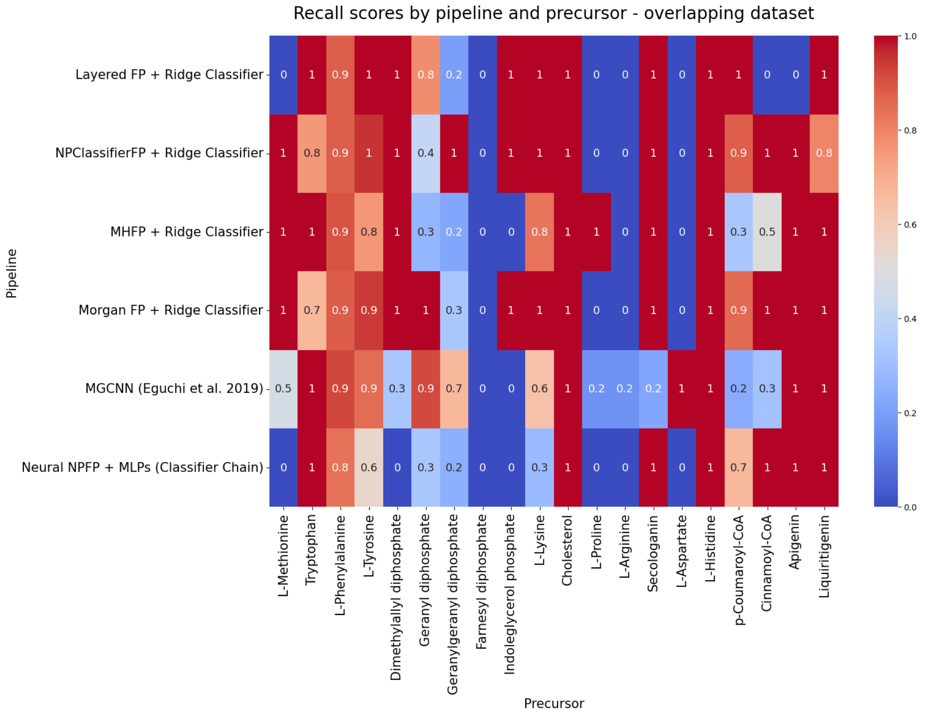


**Fig. 9.** Recall scores for each label and model

## 4 Statistical differences

In all the tables below, the asterisks are used to denote different levels of statistical significance based on p-values estimated from a Wilcoxon Rank Test. Four asterisks (****) indicate a p-value $\leq 0.0001$, signaling a highly significant result. Three asterisks (***) represent a p-value $\leq 0.001$, denoting a very significant result. Two asterisks (**) correspond to a p-value $\leq 0.01$, marking a significant result, while one asterisk (*) reflects a p-value $\leq 0.05$, indicating marginal significance.

### 4.1 Test dataset

**Table 1.** Significant differences between pipelines for F1 scores in test dataset

| Pipeline 1 | Pipeline 2 | Differences (F1 score) |
|---|---|---|
| Layered FP + Ridge Classifier | MGCNN (Eguchi et al. 2019) | * |
| Layered FP + Ridge Classifier | Neural NPFP + MLPs (Classifier Chain) | ** |
| NPClassifierFP + Ridge Classifier | Neural NPFP + MLPs (Classifier Chain) | * |

**Table 2.** Significant differences between pipelines for precision scores in test dataset

| Pipeline 1 | Pipeline 2 | Differences (Precision score) |
|---|---|---|
| Layered FP + Ridge Classifier | NPClassifierFP + Ridge Classifier | * |
| Layered FP + Ridge Classifier | MHFP + Ridge Classifier | * |
| Layered FP + Ridge Classifier | MGCNN (Eguchi et al. 2019) | ** |
| Layered FP + Ridge Classifier | Neural NPFP + MLPs (Classifier Chain) | ** |
| MHFP + Ridge Classifier | MGCNN (Eguchi et al. 2019) | * |
| Morgan FP + Ridge Classifier | MGCNN (Eguchi et al. 2019) | ** |
| Morgan FP + Ridge Classifier | Neural NPFP + MLPs (Classifier Chain) | * |

**Table 3.** Significant differences between pipelines for recall scores in test dataset

| Pipeline 1 | Pipeline 2 | Differences (Recall score) |
|---|---|---|
| Layered FP + Ridge Classifier | Neural NPFP + MLPs (Classifier Chain) | * |
| NPClassifierFP + Ridge Classifier | Neural NPFP + MLPs (Classifier Chain) | * |
| MHFP + Ridge Classifier | MGCNN (Eguchi et al. 2019) | * |
| MGCNN (Eguchi et al. 2019) | Neural NPFP + MLPs (Classifier Chain) | ** |

### 4.2 Alkaloids dataset

**Table 4.** Significant differences between pipelines for F1 scores in the alkaloids dataset

| Pipeline 1 | Pipeline 2 | Differences (F1 score) |
|---|---|---|
| Layered FP + Ridge Classifier | NPClassifierFP + Ridge Classifier | * |
| Layered FP + Ridge Classifier | MHFP + Ridge Classifier | **** |
| Layered FP + Ridge Classifier | Morgan FP + Ridge Classifier | **** |
| Layered FP + Ridge Classifier | Neural NPFP + MLPs (Classifier Chain) | **** |
| Layered FP + Ridge Classifier | MGCNN (Eguchi et al. 2019) | *** |
| NPClassifierFP + Ridge Classifier | MHFP + Ridge Classifier | * |
| NPClassifierFP + Ridge Classifier | Morgan FP + Ridge Classifier | ** |
| NPClassifierFP + Ridge Classifier | Neural NPFP + MLPs (Classifier Chain) | **** |
| NPClassifierFP + Ridge Classifier | MGCNN (Eguchi et al. 2019) | ** |
| MHFP + Ridge Classifier | Neural NPFP + MLPs (Classifier Chain) | **** |
| Morgan FP + Ridge Classifier | Neural NPFP + MLPs (Classifier Chain) | **** |
| Neural NPFP + MLPs (Classifier Chain) | MGCNN (Eguchi et al. 2019) | **** |

**Table 5.** Significant differences between pipelines for precision scores in the alkaloids dataset

| Pipeline 1 | Pipeline 2 | Differences (Precision score) |
|---|---|---|
| Layered FP + Ridge Classifier | MHFP + Ridge Classifier | ** |
| Layered FP + Ridge Classifier | Morgan FP + Ridge Classifier | * |
| Layered FP + Ridge Classifier | Neural NPFP + MLPs (Classifier Chain) | **** |
| Layered FP + Ridge Classifier | MGCNN (Eguchi et al. 2019) | **** |
| NPClassifierFP + Ridge Classifier | MHFP + Ridge Classifier | *** |
| NPClassifierFP + Ridge Classifier | Morgan FP + Ridge Classifier | *** |
| NPClassifierFP + Ridge Classifier | Neural NPFP + MLPs (Classifier Chain) | **** |
| NPClassifierFP + Ridge Classifier | MGCNN (Eguchi et al. 2019) | **** |
| MHFP + Ridge Classifier | Neural NPFP + MLPs (Classifier Chain) | **** |
| MHFP + Ridge Classifier | MGCNN (Eguchi et al. 2019) | *** |
| Morgan FP + Ridge Classifier | Neural NPFP + MLPs (Classifier Chain) | **** |
| Morgan FP + Ridge Classifier | MGCNN (Eguchi et al. 2019) | *** |

**Table 6.** Significant differences between pipelines for recall scores in the alkaloids dataset

| Pipeline 1 | Pipeline 2 | Differences (Recall score) |
|---|---|---|
| Layered FP + Ridge Classifier | NPClassifierFP + Ridge Classifier | ** |
| Layered FP + Ridge Classifier | MHFP + Ridge Classifier | *** |
| Layered FP + Ridge Classifier | Morgan FP + Ridge Classifier | *** |
| Layered FP + Ridge Classifier | Neural NPFP + MLPs (Classifier Chain) | **** |
| NPClassifierFP + Ridge Classifier | Neural NPFP + MLPs (Classifier Chain) | **** |
| MHFP + Ridge Classifier | Neural NPFP + MLPs (Classifier Chain) | **** |
| MHFP + Ridge Classifier | MGCNN (Eguchi et al. 2019) | ** |
| Morgan FP + Ridge Classifier | Neural NPFP + MLPs (Classifier Chain) | **** |
| Morgan FP + Ridge Classifier | MGCNN (Eguchi et al. 2019) | ** |
| Neural NPFP + MLPs (Classifier Chain) | MGCNN (Eguchi et al. 2019) | **** |

### 4.3 Clustered dataset

**Table 7.** Significant differences between pipelines for F1 scores in the clustered dataset

| Pipeline 1 | Pipeline 2 | Differences (F1 score) |
|---|---|---|
| Layered FP + Ridge Classifier | Neural NPFP + MLPs (Classifier Chain) | ** |
| NPClassifierFP + Ridge Classifier | Neural NPFP + MLPs (Classifier Chain) | ** |
| MHFP + Ridge Classifier | Neural NPFP + MLPs (Classifier Chain) | ** |
| Morgan FP + Ridge Classifier | Neural NPFP + MLPs (Classifier Chain) | ** |

**Table 8.** Significant differences between pipelines for precision scores in the clustered dataset

| Pipeline 1 | Pipeline 2 | Differences (Precision score) |
|---|---|---|
| Layered FP + Ridge Classifier | Neural NPFP + MLPs (Classifier Chain) | * |
| Morgan FP + Ridge Classifier | Neural NPFP + MLPs (Classifier Chain) | * |

**Table 9.** Significant differences between pipelines for recall scores in the clustered dataset

| Pipeline 1 | Pipeline 2 | Differences (Recall score) |
|---|---|---|
| Layered FP + Ridge Classifier | Neural NPFP + MLPs (Classifier Chain) | ** |
| NPClassifierFP + Ridge Classifier | Neural NPFP + MLPs (Classifier Chain) | * |
| MHFP + Ridge Classifier | Neural NPFP + MLPs (Classifier Chain) | * |
| Morgan FP + Ridge Classifier | Neural NPFP + MLPs (Classifier Chain) | ** |

### 4.4 Overlap dataset

**Table 10.** Significant differences between pipelines for F1 scores in the overlapping dataset

| Pipeline 1 | Pipeline 2 | Differences (F1 score) |
|---|---|---|
| Neural NPFP + MLPs (Classifier Chain) | MGCNN (Eguchi et al. 2019) | ** |

**Table 11.** Significant differences between pipelines for Precision scores in the overlapping dataset

| Pipeline 1 | Pipeline 2 | Differences (Precision score) |
|---|---|---|
| MHFP + Ridge Classifier | Neural NPFP + MLPs (Classifier Chain) | * |
| Morgan FP + Ridge Classifier | Neural NPFP + MLPs (Classifier Chain) | * |

**Table 12.** Significant differences between pipelines for Recall scores in the overlapping dataset

| Pipeline 1 | Pipeline 2 | Differences (Recall score) |
|---|---|---|
| NPClassifierFP + Ridge Classifier | MGCNN (Eguchi et al. 2019) | ** |
| MHFP + Ridge Classifier | MGCNN (Eguchi et al. 2019) | * |
| Neural NPFP + MLPs (Classifier Chain) | MGCNN (Eguchi et al. 2019) | ** |
